# Potential of seagrass habitat restorations as nature-based solutions: Practical and scientific implications in Indonesia

**DOI:** 10.1007/s13280-022-01811-2

**Published:** 2022-12-09

**Authors:** Husen Rifai, Jay Mar D. Quevedo, Kevin Muhamad Lukman, Calyvn F. A. Sondak, Johan Risandi, Udhi Eko Hernawan, Yuta Uchiyama, Rohani Ambo-Rappe, Ryo Kohsaka

**Affiliations:** 1 Research Center for Oceanography – National Research and Innovation Agency (BRIN), Jl. Pasir Putih 1, Ancol Timur, Jakarta, 14430 Indonesia; 2 Graduate School of Agricultural and Life Sciences, The University of Tokyo, 1-1-1 Yayoi, Bunkyo-ku, Tokyo, 113-8657 Japan; 3 Department of Marine Science, Faculty of Fisheries and Marine Science, Sam Ratulangi University, l. Kampus, Bahu, Kec. Malalayang, Manado, Sulawesi Utara 95115 Indonesia; 4 Marine Research Center, Ministry of Marine Affairs and Fisheries, Jl. Pasir Putih 1, Ancol Timur, Jakarta, 14430 Indonesia; 5 Graduate School of Human Development and Environment, Kobe University, 3-11 Tsurukabuto, Nada-ku, Kobe City, Hyogo 657-850 Japan; 6 Department of Marine Science, Faculty of Marine Science and Fisheries, Hasanuddin University, Jl. Perintis Kemerdekaan Km. 10 Tamalanrea, Makassar, 90245 Indonesia

**Keywords:** Blue carbon, Climate change mitigation, Ecosystem services, Local stakeholders, Societal challenges

## Abstract

Seagrasses offer diverse ecosystem services, yet, they are among the most threatened ecosystems. When degraded or destroyed, their services are lost or reduced in the process, affecting, for instance, local communities directly dependent on their livelihood provision. The Intergovernmental Panel on Climate Change (IPCC) reported that climate change is projected to worsen over time; thus, there is an urgent need for mitigation strategies in practice and also in the longer term. This work aims to provide an alternative perspective of seagrass restoration as a nature based solution (NbS) on a global scale, yet, giving an emphasis on tropical regions such as Indonesia. We focused on seagrass restorations which are not yet well established in comparison with other restoration programs (e.g., mangroves) despite their critical roles. We present in this work how restoring seagrass meadows fits the global standard of NbS published by the International Union for Conservation of Nature (IUCN). The results of this study can serve as a basis for promoting seagrass restorations as NbS against climate change particularly in countries with a wide extent of seagrass coverage.

## Introduction

Seagrasses are marine flowering plants found on the coasts of all continents except Antarctica (Duarte 2002). Seagrass meadows are among the most important and productive marine ecosystems globally, providing multiple and essential ecosystem services that directly or indirectly benefit people (Cullen-Unsworth et al. 2014). For instance, seagrass meadows provide a habitat for multiple life stages of commercially valuable fishes and invertebrates, support local communities as food and livelihood sources, improve water quality, stabilize sediment and prevent resuspension, and offer coastal protection services by attenuating wave and tidal current energy (e.g., Fourqurean et al. 2012; Christianen et al. 2013; Unsworth et al. 2014; Quevedo et al. 2020, 2022; McKenzie et al. 2021). Additionally, seagrasses along with mangroves and salt marshes, collectively known as blue carbon ecosystems (BCEs), have gained renewed global attention for their essential role in climate change mitigation (Duarte et al. 2013) because of their efficient sediment accumulation capacity, which makes them sequester and store organic carbon in sediments for the long term (Miyajima et al. 2021). Thus, recently, there has been an increase in scientific investigations of seagrass blue carbon, for instance, in policy and financial scheme discussions such as in carbon–neutral policies and carbon offsetting (e.g., Kuwae et al. 2022).

Despite all the essential benefits they provide at the local and global scales, there has been a decline in seagrass cover globally, which is mainly attributed to anthropogenic disturbances (Duarte 2002; Orth et al. 2006). For instance, in Southeast Asia, where seagrass diversity is considered a global hotspot, seagrass meadows are declining, with an estimated average decline of 5% per year from 2000 to 2020 due to multiple human-induced stressors such as increasing coastal populations, unregulated coastal development, unsustainable tourism industry, and destructive fishing techniques (Waycott et al. 2009; Fortes et al. 2018). Among the countries in Southeast Asia, Indonesia, which has the largest extent of seagrass meadows (Fortes et al. 2018), has seen a decline of 30–40% in seagrass cover since the 1960s caused by multiple stressors including aquaculture expansion and coastal development (Alongi et al. 2016; Unsworth et al. 2018b). When these ecosystems are degraded, their beneficial ecosystem services are reduced or lost in the process (Cullen-Unsworth et al. 2014). In Indonesia, seagrass degradation could potentially lose 5.62–8.40 tons of sequestered C ha-1 y-1 (Wahyudi et al. 2020), which is at least two-fold higher than the global average (2.78 ton C ha-1 y-1) (Duarte et al. 2013). Meanwhile, at the local level, it can potentially affect coastal communities, particularly their livelihoods (e.g., Lukman et al. 2021; Quevedo et al. 2021a). Thus, a call to improve the management, conservation, and restoration of seagrasses is urgently needed in Indonesia (Unsworth et al. 2018b; Rifai et al. 2022), which is in line with global initiatives such as United Nations (UN) Decade on Ecosystem Restoration from 2021 to 2030 (Fischer et al. 2021).

Historically, seagrass restoration efforts have been typically implemented across small spatial extents limited to a few hectares partially due to the time and money required for the methods used (Orth et al., 2006). In addition, there is also a lack of up-to-date information on the status and condition of many seagrass meadows, which are essential details to have for seagrass conservation (Unsworth et al. 2019). Seagrasses, in general, received limited attention both in scientific investigation and management agendas, and are often included with other ecosystems. For example, in the Intergovernmental Platform on Biodiversity and Ecosystem Services (IPBES 2018) regional assessment report on biodiversity and ecosystem services for Asia and the Pacific, seagrasses are given a separate focus as “seagrass beds” and “other algal communities” along with “mangroves” or “coral and other reefs.”

However, current trends of seagrass restoration and conservation are increasing partially because of the renewed interest in seagrass meadows in the blue carbon (climate change mitigation) contexts (Shilland et al. 2021), though, it is known that seagrass rehabilitation or restoration is a slow process, with years to decades to observe a successful recolonization (Leschen et al. 2010). Aside from its significant contribution to global climate change mitigation, seagrass restoration leads to the recovery of other beneficial ecosystem services such as improved water quality, increased epifauna invertebrate population, and fishery industry (Lefcheck et al. 2017; Edward 2018; Orth et al. 2020). Thus, seagrass restoration can be considered a nature-based solution (NbS), which is defined by the International Union for Conservation of Nature (IUCN 2016) as “actions to protect, manage and restore natural or modified ecosystems, that address societal challenges effectively and adaptively, simultaneously providing human well-being and biodiversity benefits.” The concept of NbS as described above, therefore, relates or overlaps with other approaches such as Ecosystem-based Adaptation (EbA), where adaptation policies and measures are geared toward the management of the natural environment (ecosystems) to reduce the vulnerability of society to climate change (Vignola et al. 2009). According to the Intergovernmental Panel for Climate Change (IPCC), the concept of EbA is similar or related to NbS (both approaches aim to manage the nature or ecosystems to produce positive outcomes—ecosystem services or benefits for society [Nesshöver et al. 2017]), however, NbS includes a broader range of approaches with safeguards, such as those that contribute to adaptation and mitigation (IPCC 2022). Thus, NbS has often been used as an ‘umbrella concept’ for these established concepts (e.g., EbA, Green–blue infrastructure, Ecosystem Approach) (Nature 2017; Nesshöver et al. 2017).

The NbS can be categorized into five main approaches including (1) ecosystem restoration, (2) issue-specific ecosystem-related, (3) ecosystem-based management, (4) ecosystem protection approaches, and (5) infrastructure-related approaches (Cohen-Shacham et al. 2016). In this paper, we specifically focused on the seagrass ecosystem restoration approach as NbS to, for instance, climate change (United Nations Environment Programme 2020). To date, applying seagrass restoration in the context of NbS is still limited globally in comparison with other ecosystems such as mangroves (e.g., Gijsman et al. 2021; Quevedo et al. 2021b). This paper aims to address this knowledge gap and provide a different perspective on seagrass restorations. Specifically, we used the global standards of NbS published by the International Union for Conservation of Nature (IUCN 2020) and provided substantial evidence on how seagrass restoration fits the criteria of IUCN’s framework (Table 1). There are eight criteria published by the IUCN (2020), however, we only focused on criterions one to five since we aim to provide conceptual evidence of seagrass restoration as NbS. The other three criteria (criterions 6 to 8) are related to implementation and mainstreaming actions (Quevedo et al., 2021b). We envisage that by providing NbS perspectives of seagrass restorations, the approach will be more appealing to stakeholders and policymakers, especially in the context of climate change mitigation and adaptation through carbon sequestration and other valuable ecosystem services (e.g., Wahyudi et al. 2020; Quevedo et al. 2022).

**Table 1:** The eight criteria of the global standard of nature-based solutions (NbS) (IUCN 2020)

Criteria	Description
1	NbS effectively address societal challenges
2	The design of NbS is informed by scale
3	NbS result in a net gain in biodiversity and ecosystem integrity
4	NbS are economically viable
5	NbS is based on inclusive, transparent, and empowering governance processes
6	NbS equitably balance trade-offs between the achievement of their primary goal(s) and the continued provision of multiple benefits
7	NbS are managed adaptively, based on evidence
8	NbS are sustainable and mainstreamed within an appropriate jurisdictional context

## Criterion 1: Seagrass restorations address societal challenges

The first criterion ensures that NbS effectively addresses societal challenges, understands clearly these challenges, and delivers substantive benefits to human well-being in response to these challenges (IUCN 2020). There are several major societal challenges mentioned by the IUCN (2016) such as climate change mitigation and adaptation, disaster risk reduction, and economic and social development. Climate change has become an increasingly discussed issue due to its impact on humans, and many climate change mitigation efforts have been carried out by optimizing the utilization of resources on land and sea (Dewi et al. 2020). Conservation and restoration of BCEs, such as seagrass meadows, are excellent examples of NbS to achieve sustainable development goals (SDGs) of climate action (Herr and Landis 2016; Fauzi et al. 2021).

Seagrasses potentially mitigate the impacts of climate change, for instance, they could reduce the impact of sea-level rise, and have a great ability to lower sea surface temperature (Rustam et al. 2017). Additionally, these habitats have a high potential as large organic carbon sinks because of their efficient sediment accumulation capacity, which makes them sequester and store organic carbon in sediments for the long term (Miyajima et al. 2021). However, there are countries (e.g., Indonesia, the Philippines) where communities are less aware of the role of seagrass meadows in climate change mitigation (Lukman et al. 2021; Quevedo et al. 2021a). In greater Southeast Asia, there is the challenge of socio-economic and cultural disconnect for the seagrass ecosystems due to the lack of appreciation and understanding of seagrass utilization and value (Fortes et al. 2018). Thus, capacity building of coastal communities to increase the awareness and utilization of seagrass ecosystem services, which is beneficial for the socio-economic aspects (e.g., fishing and gleaning livelihood source, tourism potential) (Quevedo et al. 2022), will be needed as an integral part of the NbS management.

Indonesia is one of the countries with expansive seagrass habitats; enabling them to store a significant portion of the world’s blue carbon, thus, these important habitats will significantly assist the country’s mitigation efforts in reducing the impact of climate change (Rifai et al. 2022). To date, the state of Indonesia’s seagrass compromises both resilience to climate change and ecosystem services provision (Unsworth et al. 2018a). A science-backed or evidence-based plan for restoring degraded BCEs will build climate change resilience and improve livelihoods (Murdiyarso et al. 2018). For example, a low-carbon development agenda consisting of blue carbon development programs and governance support could unlock the social, environmental, and economic benefits of blue carbon (Murdiyarso et al. 2015). Moreover, the inclusion of blue carbon in the Paris Agreement has created a platform for Indonesia to put coastal conservation at the heart of climate mitigation (Unsworth et al. 2018a). However, to date, there are no specific laws and/or regulations in Indonesia dedicated to seagrass ecosystems (Rifai et al. 2022), despite the country’s recognition to manage and conserve carbon sinks (Stankovic et al. 2022). Nevertheless, a new set of regulations with guidelines and plans for carbon trading to achieve their nationally determined contribution (NDC) (Situmorang and Putri 2022) is currently under preparation (Stankovic et al. 2021). As described in Presidential Regulation No. 98 of 2021, the carbon trading scheme will cover energy, agriculture, forestry, and other sectors (Situmorang and Putri 2022). This progress can open opportunities for the implementation of seagrass restoration in Indonesia as NbS to address the impacts of climate change.

## Criterion 2: Seagrass restorations can cover a wide scale

Among the criteria, this criterion is complex since it discusses that NbS designs should (a) recognize and respond to interactions between economy, society, and ecosystems, (b) integrate with other complementary interventions and promote synergies across sectors, (c) and incorporate risk identification and risk management beyond the intervention site (IUCN 2020). The review article of Tan et al. (2020) presents a good example of how seagrass restoration fits the second criteria of NbS. For instance, they presented pre-restoration considerations including (1) clear accountability and (2) adequate resourcing and strategic prioritization of efforts, which we cautiously interpreted as design considerations before implementing NbS. In the first consideration, Tan et al. (2020) highlighted that enabling policies and legislations will facilitate broad-scale seagrass restoration efforts; citing the Catchment Management Framework in Victoria, Australia, which incorporates environmental, economic, and social considerations for coordinated management, as an example. In the second consideration, they further emphasized the importance of identifying the intervention site, where synergies among sectors (e.g., social, environmental, and cost) are possible. Moreover, Tan et al. (2020) discussed that restoration programs should be holistic and cover a broader scale (e.g., whole landscape and associated benefits) rather than a single entity.

The study of Valdez et al. (2020) is another example of how seagrass restoration if implemented in the context of NbS meets the second criterion. They reviewed and documented the beneficial effects of integrating positive species interactions in seagrass restoration designs. For instance, in long-distance facilitations, which occur when seagrasses are benefited by other species that are in adjacent areas or not in direct contact (e.g., mangroves), challenges in seagrass restoration such as light limitation and nutrient stress can be mitigated; mangroves can remove particulates from the water, thus, help in addressing light available for seagrasses (van de Koppel et al. 2015). Valdez et al. (2020) noted that integrating long-distance facilitation into site selection for restoration projects will likely result in a positive outcome, though further research is needed.

Seagrass restoration helps protect tropical beaches from erosion (James et al. 2019), which in turn protects nearby physical assets (e.g., hotels, beachfront pavements) and the economy (e.g., tourism industry). Thus, nature-based foreshore stabilization can be implemented as a long-term solution in coastal areas with high erosion rates, as documented in the study conducted by James et al. (2019). Their study showed the long-term efficacy of nature-based beach management by evaluating coastal areas with vegetation and without vegetation. In Indonesia, coastal erosion hinders the socio-economic development of coastal zones such as on the northern coast of Java Island. Implementing seagrass restoration is an alternative to traditional hard-built structures (e.g., seawalls, jetties), and it can be a more sustainable and resilient long-term solution (Solihuddin et al. 2021). Alternatively, seagrass restoration can be applied together with existing hard-built infrastructures. The presence of coastal vegetations at the offshore side of the infrastructures dissipate wave energy, which in turn reduces the load to built structures, thereby prolonging the service and protection of other physical assets (Vuik et el. 2016).

## Criterion 3: Seagrass restorations can result in biodiversity gain and ecosystem integrity

This criterion sets guidelines that NbS result in a net gain to biodiversity and ecosystem integrity (IUCN 2020). The design and implementation of NbS must consider the integrity of the target ecosystem and proactively seek to enhance the connectivity with other ecosystems. Furthermore, by identifying clear and measurable biodiversity outcomes, NbS can set targets for conservation activities. There are existing studies that documented increased biodiversity following a successful seagrass restoration program. For instance, the study conducted by Edward (2018) in the Gulf of Mannar, India showed that the population of associated organisms such as fish and other macrofauna was proportional to the increase of seagrass cover. Similarly, Lefcheck et al. (2017) observed that after less than a decade of seagrass restoration in the coastal bays of the midwestern Atlantic, USA, the invertebrate community became richer and exhibited greater variation in functional traits resulting from the increasing density of eelgrass. Additionally, successful seagrass restoration programs have led to ecosystem integrity with evidence showing a rapid recovery of seagrass ecosystem services (Orth et al. 2020).

One of the indicators to develop NbS under this criterion is that the current state of seagrass meadows in the target site should be well established (IUCN 2020). This becomes a challenge since the global distribution and status of seagrass meadows are difficult to monitor and map, and there are still regions with seagrass meadows remaining to be explored (Unsworth et al. 2019). However, studies and techniques on seagrass mapping are advancing and more regions are now being identified and quantified. For instance, McKenzie et al. (2022) mapped the seagrass cover of the Great Barrier Reef using combined methods of field-based in situ mapping, high earth boundary tracking, high earth mapping with unoccupied aerial systems, and satellite-capture imagery mapping. These technologies when combined produce more accurate seagrass maps. Similarly, Nguyen et al. (2022) used combined methods of in situ mapping using handheld devices and object-based classification mapping (remote sensing) to produce high-resolution seagrass map images of Nam Yet Island, Vietnam. Recently, unmanned aerial vehicles (UAVs) or drones and deep learning techniques have been used and proven to obtain higher-resolution images for seagrass mapping (e.g., Tahara et al. 2022). These advancements suggest that seagrasses are currently receiving more scientific and practical attention compared to earlier periods.

In Indonesia, the nationwide mapping of seagrass meadows conducted by Hernawan et al. (2021) has paved the way for other Indonesian scientists to strategically identify areas across the country that need restoration projects, particularly those pressured by anthropogenic activities. For instance, in Bontang, East Kalimantan province, seagrass meadows are severely damaged by fishing activities, which in turn affects the livelihoods of small-scale fishers (Irawan et al. 2019). In Karimunjawa Island, Central Java province, pollution discharge from domestic wastes threatens seagrass meadows as perceived by coastal communities (Quevedo et al. 2021a). A similar scenario was observed in Spermonde Archipelago, South Sulawesi province, where seagrasses’ condition was affected by the nutrient loading, turbidity, and total suspended solids resulting from domestic solid and liquid wastes, which prompted a restoration project in 2016 (Ambo-Rappe 2022).

As NbS strives to enhance the connectivity between ecosystems, this implies the notion of seagrasses as part of a larger ecosystem (e.g., along with coral reefs and mangroves). Such connectivity can be done to resolve existing challenges, such as the case observed in Wakatobi National Park, Indonesia, where initiatives of fruit tree plantations and local ecological knowledge to identify threats to seagrass and educational programs facilitated the stabilization of river banks and reducing sediment deposition to the coast (Unsworth et al. 2019). The notion of people’s involvement in seagrass restoration activities to enhance biodiversity is another important aspect of NbS, which will be further elaborated in Criteria 5.

## Criterion 4: Seagrass restorations are economically viable

This criterion emphasizes that NbS should be economically feasible to be conducted through the support of financial institutions and/or incentive schemes (IUCN 2020). There are several studies documenting that coastal ecosystem restorations including seagrasses are economically viable as they provide net benefits, which is defined as the monetary value of ecosystem services generated by the restored ecosystem (Stewart-Sinclair et al. 2021).

Seagrass restoration has been conducted to recover ecosystem services or benefits lost due to habitat degradation (Rezek et al. 2019). Multiple co-benefits obtained from a restored habitat could highlight the economic viability of seagrass restoration projects and encourage other groups to conduct such activities. For instance, in Australia, recovery of ecosystem functions through seagrass restoration can potentially produce net benefit ranging from AUD 40 000 ha^−1^ to AUD 7.8 million ha^−1^ (Rogers et al. 2019). In Virginia, USA, a 7-km^2^ of restored eelgrass has removed 9,600 tons of CO2 from the atmosphere over 15 years which is equivalent to financial benefits of as much as US$ 87 000 or about US$ 124 ha^−1^ (Oreska et al. 2020). In Atlantic coastal lagoons, after 10 years, well-developed restored seagrass meadows provide important multiple co-benefits such as housing diverse animal communities, sequestering substantial stocks of carbon and nitrogen, and facilitating the restoration of previously depleted seagrass associated fauna “the bay scallops” (Orth et al. 2020). However, despite the economic viability of restored seagrass meadows, it is widely recognized that their re-establishment are time consuming, frequently requiring years to decades (Tan et al. 2020). Meanwhile, in areas where seagrass restoration is perceived as a way to promote a state shift, from an unvegetated to a vegetated state, coastal managers and practitioners should understand and consider the factors limiting the transition to meet their expectations and restoration goals (Paulo et al. 2019).

The cost for seagrass restoration is approximately US$ 700 000 ha^−1^, which is much lower than the cost for coral reef restoration which is nearly US$ 3 000 000 ha^−1^ (Bayraktarov et al. 2015). Moreover, under a best scenario, seagrass restorations can yield a positive internal rate of returns (IRR) of 3% and a cost–benefit ratio of 1.7, indicating that the benefit exceeded the cost (de Groot et al. 2013). In this case, it has been calculated the time to return on investment for seagrass restoration is more than 70 years (Stewart-Sinclair et al. 2021). This longer time frame could be an important predictor of a net benefit in seagrass restoration, however, the future cost would decline after the installation of the restoration project, and a minimum amount will be required for maintenance and monitoring (de Groot et al. 2013).

The restoration cost can also be influenced by the selected restoration method and the addition of advanced equipment involved in setting up seagrass restoration projects. For instance, in Australia, mechanical seagrass plantation such as the use of an ecosub system (a mechanical device used to cut and plant large sods) will cost AUD 1 000 000 ha^−1^, covering the design and development, fabrication, testing, and site selection (Paling et al. 2009). In contrast, manual plantation will only cost AUD 16 000–34 000 ha^−1^ if conducted by volunteers and AUD 84 000–168 000 ha^−1^ if the restoration is conducted by professionals (Paling et al. 2009). The selection between seed- or transplant-based methods for seagrass restoration is also an important factor in determining the length of maintenance and monitoring period of the restoration site. When seeds are used for seagrass restoration, it is estimated to take only 10 years to recover in comparison with natural recovery which takes 100 years (Reynolds et al. 2016). For example, a large-scale seed-based seagrass restoration has been conducted in midwestern Atlantic coastal lagoons, leading to a rapid recovery of the previously degraded seagrass bed after 10 years (Orth et al. 2020). However, using seeds and seedlings in seagrass restorations can be challenging, especially when the sites are exposed to, for instance, high wave energy (Paling et al. 2009) or the presence of seed predators such as shore crabs, hermit crabs, and sea urchins (Infantes et al. 2016). Thus, site selection for seagrass transplantation using seeds should be properly observed to ensure the successful growth of the planted seeds.

In Indonesia, a long-term seagrass restoration has been conducted in the Spermonde region, South Sulawesi using multiple seagrass species in a 600-m^2^ area. The project, which started with roughly 10% of seagrass transplant coverage, had a total cost of approximately US$ 100 000 covering the preparation cost, initial installation, and a 3-year monitoring program. A successful rate was obtained after 7 years through the indication of an increased cover of restored seagrass meadows, more diverse faunal communities, and increased coastal protection from erosion (Asriani et al. 2019).

## Criterion 5: Seagrass restorations empower local stakeholders

The fifth criterion of the global standard of NbS states that “NbS acknowledges, involves and responds to the concerns of a variety of stakeholders, especially rights holders” (IUCN 2020, p. 14). This criterion has five indicators including (1) feedback and grievance resolution mechanism, (2) participation is based on mutual respect, (3) stakeholders have been identified and involved, (4) collaborative decision-making process, and (5) respect boundaries and enable joint decision-making (IUCN 2020). Seagrass restorations fit this criterion well since ensuring the successful implementation of this activity requires a great understanding of ecological science and a comprehensive approach toward the integration of human participation into all stages of restoration measures (Wylie et al. 2016). Engaging local communities residing adjacent to seagrass areas in all restoration activities is an integral part of seagrass restoration projects. This is because the fundamental objectives of ecosystem restoration such as seagrass restoration include the recovery of degraded habitats to support biodiversity and providing various goods and services to local people (Fischer et al. 2021). Moreover, seagrass restoration projects can be considered successful if local communities provide full support to the project implementation (Bennett and Dearden 2014).

Given that seagrass restoration projects have high labor costs since many people are needed to collect restoration materials and deploy the transplant units to the restoration site, the involvement of communities (citizen scientists) and volunteers will significantly reduce this cost (Tan et al. 2020). In addition, there are other benefits of engaging local community members in seagrass restoration activities. First, involving local communities in all stages of restoration efforts will generate a sense of ownership and encourage community members to return and provide more of their time (Tanner et al., 2014). Second, citizen scientist participation will allow a larger and longer-term data collection leading to a greater understanding of the seagrass life cycle (Jones et al. 2018). Third, local community involvement will allow rapid knowledge transfer from seagrass scientists to community members which is very useful to increase the understanding of seagrass-related matters among the people (Tanner et al. 2014; Jones et al. 2018).

Unlike mangrove or coral restoration programs, however, there is no incentive from the Indonesian government to foster community involvement in seagrass restoration projects. Moreover, Rifai et al. (2022) noted that the participation of local stakeholders in seagrass restoration activities is hindered by their lack of awareness and appreciation of the functions and services of seagrass habitats. The lack of awareness and appreciation is considered the biggest threat to seagrass conservation (Unsworth et al. 2019). Thus, there is a need to enhance the awareness to foster active participation of the people in seagrass restoration activities.

## Potential implementation of seagrass restoration in the context of NbS in Indonesia

Under the Paris Agreement in 2015, Indonesia has committed to reducing greenhouse gas emissions by 29% with the national budget, and up to 41% with global support by 2030 (Murdiyarso et al. 2018). Following this commitment, Indonesia has proposed a plan to include blue carbon in its NDC at the Conference of the Parties (COP) 22 meeting. Then, at COP 25, Indonesia has clearly stated to include blue carbon ecosystems such as seagrass and mangrove ecosystems as a means to address climate change. However, Indonesia’s seagrass meadows are currently in moderate condition, allowing opportunities to be improved (Hernawan et al. 2021). The declining condition of seagrass meadows in the country suggests that restoration programs are urgently needed.

Seagrass restoration programs in Indonesia, in general, are limited (Asriani et al. 2018), about 22 restoration projects recorded from 1987 to the present, despite the urgency to restore seagrass meadows in the country (Hernawan et al. 2021; Rifai et al. 2022). Additionally, the outcome of these projects is not well documented since publications of results or project reports were limited to a few (Williams et al. 2017; Ambo-Rappe 2022). There is also the concern about the lack of restoration activities due to the relatively lesser attention given to seagrasses compared with other ecosystems such as mangroves (Nadiarti et al. 2012). We argue that there is a need to revisit how Indonesia can implement seagrass restoration programs that can produce similar trends observed in mangrove restoration activities. One approach is introducing seagrass restoration in the context of NbS particularly in addressing climate change (UNEP 2020). There is an opportunity in Indonesia to implement seagrass restoration in the context of NbS to meet their national commitment to reducing greenhouse gas emissions.

In this paper, we presented a different perspective of seagrass restorations. Interpreting the five criteria of the global standard of NbS (IUCN 2020), we summarized and reflected here on the potential of seagrass restoration as NbS in Indonesia. The country has a wide extent of seagrass cover, enabling them to store a significant amount of the world’s blue carbon, thereby putting them on the global map as an important ally to climate change mitigation (Hernawan et al. 2021; Rifai et al. 2022). Recent estimates showed that Indonesia’s seagrass meadows can store up to 368.5 Tg C or about 1.7% of the total blue carbon reservoir in the world (Alongi et al. 2016). Using the US standard of carbon pricing at $ 10 ton-1 in 2016 (Hamrick and Gallant 2017), it is estimated that the value of Indonesia’s seagrass meadows related to carbon stock services (Alongi et al. 2016) is approximately US$ 3 685 000 000. As we carefully provided evidence that seagrass restorations are economically viable (see Criterion 4), we envisage that this new perspective will attract international organizations (e.g., Conservation International) and investors (e.g., World Bank) to consider utilizing seagrass restorations as NbS to address global climate change through, for example, voluntary carbon market schemes (Shilland et al. 2021). This scheme, which refers to voluntary payments made by individuals or organizations from regional to global based on a chosen carbon standard (Shilland et al. 2021), can be a reliable source of funds to ensure the sustainability of seagrass restoration projects in Indonesia. This will be in line with the NDC goal of Indonesia as the government strongly encourages the implementation of the carbon tax (Situmorang and Putri 2022), though, currently, the Ministry of Finance through the Fiscal Policy Agency (BKF) is still preparing the roadmap to implement this concept and discussing the potential of implementation with House of Representative members of Indonesia.

## Conclusion

We presented here conceptual evidence on how seagrass restoration meets the first five criteria of NbS proposed by IUCN (2020), namely (1) addressing societal challenges, (2) covering a large scale, (3) recovering biodiversity loss, (4) economically viable, and (5) empowering local societies. Looking at Indonesia’s case, there is a potential for implementing seagrass restoration in the context of NbS. However, the lack of the national budget and low awareness of the communities regarding the importance of seagrass restoration can hinder the implementation of this concept. To address these issues, we argue that all stakeholders (e.g., the scientific community, government, local communities, and non-government organizations) need to collaborate to solve the underpinning problems effectively; capacity building and seagrass awareness campaigns, efficient and realistic restoration designs, and implementation of payment for ecosystem services. By implementing seagrass restorations in the context of NbS, we envisage that it will attract more restoration activities in Indonesia compared to the present, thereby, degraded seagrass ecosystems will recover quickly, allowing them to provide maximum ecosystem services that are integral in the era of climate change.
