# Correction to: Potential of seagrass habitat restorations as nature-based solutions: Practical and scientific implications in Indonesia

**DOI:** 10.1007/s13280-023-01916-2

**Published:** 2023-09-08

**Authors:** Husen Rifai, Jay Mar D. Quevedo, Kevin Muhamad Lukman, Calyvn F. A. Sondak, Johan Risandi, Udhi Eko Hernawan, Yuta Uchiyama, Rohani Ambo-Rappe, Ryo Kohsaka

**Affiliations:** 1 Research Center for Oceanography – National Research and Innovation Agency (BRIN), Jl. Pasir Putih 1, Ancol Timur, Jakarta, 14430 Indonesia; 2 Graduate School of Agricultural and Life Sciences, The University of Tokyo, 1-1-1 Yayoi, Bunkyo-ku, Tokyo, 113-8657 Japan; 3 Department of Marine Science, Faculty of Fisheries and Marine Science, Sam Ratulangi University, l. Kampus, Bahu, Kec. Malalayang, Manado, Sulawesi Utara 95115 Indonesia; 4 Marine Research Center, Ministry of Marine Affairs and Fisheries, Jl. Pasir Putih 1, Ancol Timur, Jakarta, 14430 Indonesia; 5 Graduate School of Human Development and Environment, Kobe University, 3-11 Tsurukabuto, Nada-ku, Kobe City, Hyogo 657-850 Japan; 6 Department of Marine Science, Faculty of Marine Science and Fisheries, Hasanuddin University, Jl. Perintis Kemerdekaan Km. 10 Tamalanrea, Makassar, 90245 Indonesia

**Correction to: Ambio (2023) 52:546–555** 10.1007/s13280-022-01811-2

The article “Potential of seagrass habitat restorations as nature-based solutions: Practical and scientific implications in Indonesia”, written by Husen Rifai, Jay Mar D. Quevedo, Kevin Muhamad Lukman, Calyvn F. A. Sondak, Johan Risandi, Udhi Eko Hernawan, Yuta Uchiyama, Rohani Ambo-Rappe and Ryo Kohsaka, was originally published Online First without Open Access. After publication in volume 52, issue 3, page 546–555, the author decided to opt for Open Choice and to make the article an Open Access publication. Therefore, the copyright of the article has been changed to © The Author(s) 2022 and the article is forthwith distributed under the terms of the Creative Commons Attribution 4.0 International License, which permits use, sharing, adaptation, distribution and reproduction in any medium or format, as long as you give appropriate credit to the original author(s) and the source, provide a link to the Creative Commons licence, and indicate if changes were made. The images or other third party material in this article are included in the article's Creative Commons licence, unless indicated otherwise in a credit line to the material. If material is not included in the article's Creative Commons licence and your intended use is not permitted by statutory regulation or exceeds the permitted use, you will need to obtain permission directly from the copyright holder. To view a copy of this licence, visit http://creativecommons.org/licenses/by/4.0/.

The original article has been corrected.

